# The Heterogeneity and Function of Stromal Cells in the Tumor Microenvironment

**DOI:** 10.34133/research.1183

**Published:** 2026-03-09

**Authors:** Xuehai Wang, Songlin Li, Yang Xue, Xiao Liang, Xuelei Ma, Kun Zhang, Ying Wang

**Affiliations:** ^1^ Department of Thoracic Surgery, Sichuan Academy of Medical Sciences and Sichuan Provincial People’s Hospital, University of Electronic Science and Technology of China, Chengdu, Sichuan Province, China.; ^2^ Department of Oncology, Sichuan Academy of Medical Sciences and Sichuan Provincial People’s Hospital, University of Electronic Science and Technology of China, Chengdu, Sichuan Province, China.; ^3^ Department of Biotherapy, State Key Laboratory of Biotherapy, West China Hospital, Sichuan University, Chengdu, Sichuan Province, China.

## Abstract

The tumor microenvironment is a central determinant of cancer progression, metastatic spread, and therapeutic resistance. Once considered passive scaffolds, stromal cells are now recognized as heterogeneous, active regulators of tumor behavior. Recent single-cell and spatial multiomics studies have resolved functionally distinct stromal subtypes, each defined by characteristic molecular programs and spatial niches, with specialized roles in extracellular matrix remodeling, immune evasion, and angiogenesis. This review synthesizes evidence for bidirectional stromal–tumor cross-talk in which cytokine networks (e.g., transforming growth factor-beta, interleukin-6, and C-X-C motif chemokine ligand 12/C-X-C chemokine receptor 4) coordinate epithelial–mesenchymal transition, stemness, chemotaxis, and vascular remodeling. Building on these insights, this review also argues for subtype-specific biomarkers and multimodal therapeutic strategies to overcome stromal-mediated resistance. Integrating stromal heterogeneity into precision-oncology workflows through standardized, lineage-resolved profiling and real-time biomarker guidance will be essential for diagnostic refinement and personalized treatment.

## Introduction

Stromal cells, once viewed as passive structural components, are now recognized as active regulators of cancer progression within the tumor microenvironment (TME) [1]. Single-cell spatial multiomics have delineated 4 conserved cancer-associated fibroblast (CAF) subtypes with distinct niches and functions. Tumor-proximal S1-CAFs drive extracellular matrix (ECM) remodeling and immunosuppression through collagen type I alpha 1 chain (COL1A1), actin alpha 2 (ACTA2), and transforming growth factor-beta 1 (TGF-β1). S2-CAFs, enriched in stromal regions, secrete interleukin-6 (IL-6) to drive inflammation. Perivascular S3-CAFs mediate stromal–immune cross-talk via platelet-derived growth factor receptor (PDGFR) and matrix metalloproteinase 2 (MMP-2). S4-CAFs within tertiary lymphoid structures (TLSs) modulate adaptive immunity via human leukocyte antigen (HLA) class II molecules and chemokines such as C-X-C motif chemokine ligand 9 (CXCL9) and C-C motif chemokine ligand 21 (CCL21) [2].

Beyond CAFs, hepatic stellate cells (HSCs) in hepatocellular carcinoma (HCC) exhibit plasticity between tumor-promoting myofibroblastic (myHSC) and cytokine-secreting inhibitory (cyHSC) states [3]. Stromal–tumor cross-talk is exemplified by semaphorin 3C (SEMA3C) from HCC cells activating HSCs via neuropilin-1 (NRP1) and integrin β1 (ITGB1), inducing IL-6 release and cholesterol biosynthesis. Conversely, CAF-derived TGF-β1 up-regulates SEMA3C in cancer stem cells (CSCs), establishing a feed-forward protumorigenic circuit that contributes to therapy resistance, including sorafenib-resistant HCC [4].

Traditional bulk omics approaches obscure stromal heterogeneity and spatial context. In contrast, single-cell and spatial multiomics, such as graph-based integration of gene expression, spatial coordinates, and molecular interactions, enable precise alignment of transcriptomic, proteomic, and spatial layers to define stromal subtypes, map cell–cell communication, and resolve niche-specific behaviors [5]. These methods reveal spatially organized stromal-immune zones and context-dependent functions, critical for understanding tumor progression.

This review therefore catalogs stromal subtypes, elucidates their mechanisms, maps spatial niches, and translates these insights into clinical strategies. We further evaluate emerging stromal-targeted therapies, propose new avenues for intervention, and highlight prognostic stromal signatures as biomarkers for precision oncology.

## Discovery of Stromal Cell Heterogeneity through Single-Cell Spatial Multiomics

### Core technologies and principles

Spatial transcriptomics platforms such as 10x Genomics Visium and NanoString GeoMx Digital Spatial Profiler enable high-resolution gene-expression profiling in intact tissues, addressing the loss of spatial context inherent to single-cell RNA sequencing (scRNA-seq). Visium provides transcriptome-wide, barcoded capture suitable for unbiased discovery of stromal subtypes, e.g., IL-6-expressing S2-CAFs, and for mapping their distribution relative to tumor nests or TLSs [6]. In contrast, GeoMx performs immunohistochemistry-guided, region-of-interest profiling of ~50 to 1,800 genes/proteins, offering high sensitivity for validating low-abundance stromal markers (e.g., CXCL9 in S4-CAFs) within defined niches [2].

Single-cell proteomics platforms, including mass cytometry (CyTOF) and multiplexed ion beam imaging by time-of-flight (MIBI-TOF), complement spatial transcriptomics by quantifying protein expression and post-translational modifications [7]. CyTOF enables high-parameter phenotyping to resolve functionally distinct stromal subsets (e.g., profibrotic versus immunosuppressive HSCs), whereas MIBI-TOF affords subcellular spatial resolution to visualize protein colocalization and cell–cell interactions such as HLA-DR^+^ S4-CAFs engaging T cells within lymphoid structures [8,9]. Computational integration using tools such as Seurat and stKeep aligns transcriptional, proteomic, and spatial coordinates to define stromal subtypes by gene programs, functional protein activity, and anatomical location, thereby enabling precise mapping of cell–cell communication and niche-specific behaviors [10].

Despite their transformative impact, single-cell and spatial multiomics technologies have important limitations that can bias stromal subtype inference and niche interpretation. For scRNA-seq, tissue dissociation itself can induce artifactual stress-response programs and alter cell-state-defining transcripts, potentially confounding cytokine-axis attribution or stromal activation signatures [11]. In solid tumors, dissociation conditions (e.g., collagenase) can trigger conserved stress responses, and cold-active protease protocols reduce these dissociation-associated transcriptional artifacts, underscoring the need for standardized processing when comparing stromal states across cohorts [12]. Droplet-based assays are additionally affected by ambient RNA contamination, which can create misleading marker detection in stromal and immune compartments unless explicitly modeled and removed [13]. For spatial transcriptomics, a major constraint of widely used spot-based platforms is multicell mixing within each spatial unit, which can blur tumor–stroma boundaries and inflate apparent stromal–immune coexpression; deconvolution methods such as RCTD and cell2location can partially recover cell-type composition but remain sensitive to reference quality and cross-platform effects [14]. More broadly, spatially resolved transcriptomics involves trade-offs between sensitivity, transcriptome coverage, and resolution, and field-facing discussions highlight these constraints as central to experimental design [15].

### Applications of multiomics in stromal cell research

**Fig. 1.**
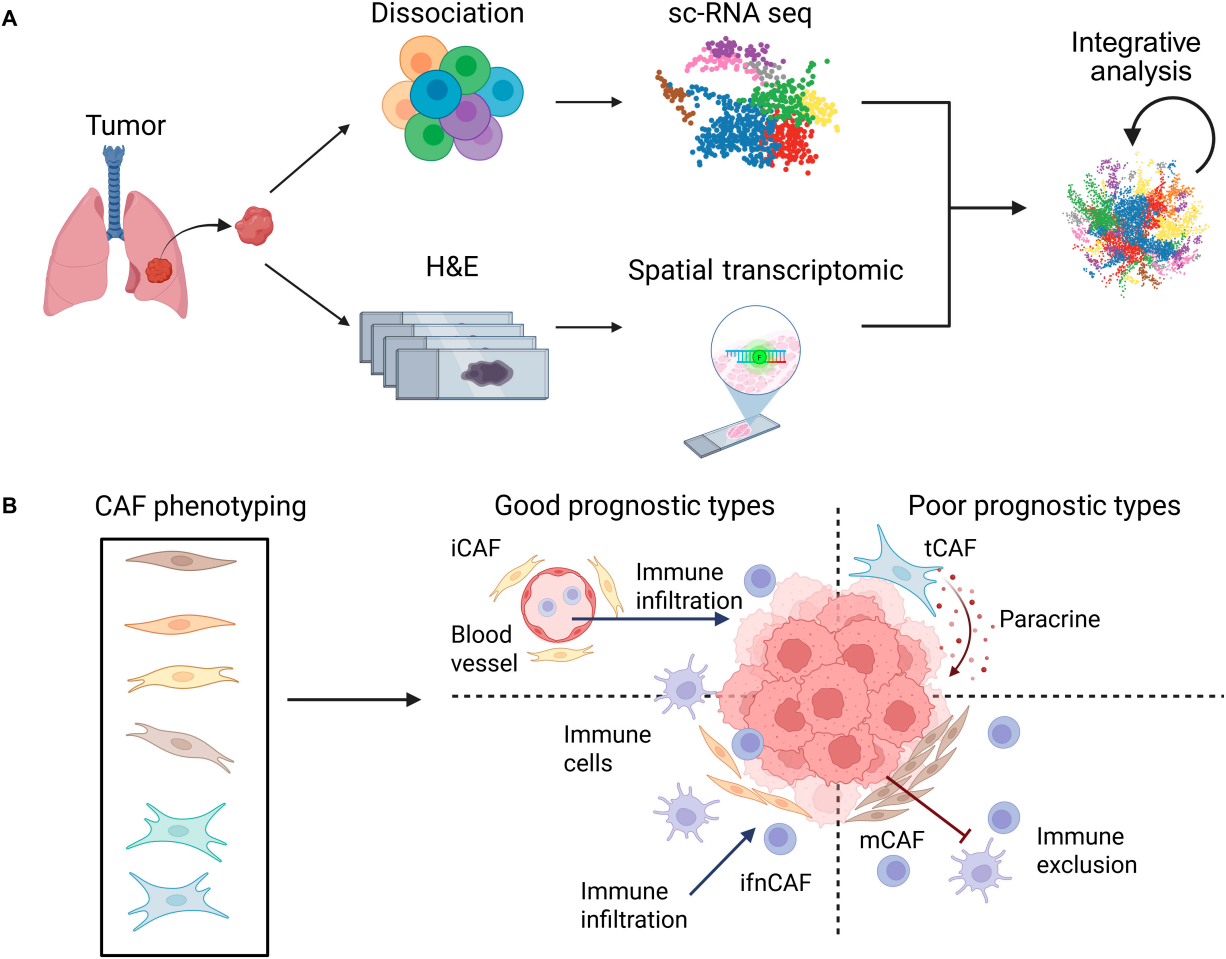
Single-cell spatial multiomics workflow for decoding stromal cell heterogeneity in TME. (A) Integrative analysis of single-cell RNA sequencing (scRNA-seq) and spatial transcriptomics from tumor tissue. (B) Phenotyping of cancer-associated fibroblasts (CAFs) and their roles in tumor microenvironment immune regulation. ifnCAF, interferon-response CAF; tCAF, tumor-like CAF; iCAF, inflammatory CAFs; mCAF, matrix CAF.

A long-standing challenge in stromal biology is the in vitro*–*in vivo disconnect. Stromal cells cultured in conventional 2-dimensional/3-dimensional (2D/3D) systems frequently lose native phenotypes and functional networks, thereby compromising their relevance to actual tumor biology [16]. Figure 1 contextualizes how multiomics addresses this issue: Fig. 1A details the workflow of integrating scRNA-seq from dissociated tumor cells and spatial transcriptomics from hematoxylin and eosin (H&E)-stained tissue sections to characterize stromal cells within the intact TME; Fig. 1B depicts CAF phenotyping into “good prognostic” (e.g., inflammatory cancer-associated fibroblast [iCAF] and interferon-response cancer-associated fibroblast [ifnCAF]) and “poor prognostic” (e.g., tumor-like cancer-associated fibroblast [tCAF] and matrix cancer-associated fibroblast [mCAF]) subtypes, alongside their roles in tumor–immune interactions (e.g., immune infiltration vs. exclusion). Leveraging single-cell spatial multiomics enables detailed TME characterization to guide the development of physiologically representative in vitro models [17].

Another well-documented issue is culture-induced convergence driven by nonphysiologic mechanics. Under standard tissue-culture conditions, patient-derived CAFs rapidly lose tumor-specific subset distinctions and converge toward a wound-healing/myofibroblastic state within a few passages [18]. This drift is exacerbated by the supraphysiologic stiffness of tissue-culture polystyrene, which pushes fibroblasts toward activation programs [19]. Conversely, microengineered niches (e.g., micropatterning and tunable-stiffness hydrogels) can re-establish in vivo-like CAF programs, yielding desmoplastic versus inflammatory phenotypes and improving drug-response fidelity [20]. Consistent with these findings, epithelial cues are required to sustain specific CAF states in pancreatic ductal adenocarcinoma (PDAC). Epithelial mitogen-activated protein kinase (MAPK)–TGF-β signaling and epidermal growth factor receptor (EGFR)/ERBB2 activation help maintain myofibroblastic CAF programs, underscoring why monoculture conditions lacking these signals promote dedifferentiation [21,22].

Multiomics also uncovers stromal-immune mechanisms that are absent in vitro. Immunosuppressive CAF functions are often lost under conventional culture conditions due to the lack of spatial context. MIBI-TOF revealed that in vivo, S1-CAFs deposit dense collagen (COL1+) and express programmed death-ligand 1 (PD-L1), forming both a physical and inhibitory barrier to T cells [23,24]. In vitro, these traits are diminished. Incorporating ECM scaffolds and vascular endothelial cells into 3D cocultures restores PD-L1 expression and immunosuppressive functionality, as confirmed via CyTOF analyses [25,26].

Multiomics has fundamentally revised our understanding of stromal plasticity. By integrating single-cell transcriptomics with tissue- and protein-level readouts, recent atlases resolve HSCs not as a uniformly myofibroblastic fate but as a conserved activation continuum that bifurcates into 2 in vivo-stable states: a matrix-producing, TGF-β/SMAD-driven myofibroblastic program (myHSC; αSMA/ACTA2^+^ and COL1A1^+^) and a cytokine-secreting, tumor-constraining program (cyHSC; e.g., IL-10^+^ and TGFB1^−^) [27]. This multiomics framework reframes experimental design and identifies therapeutic opportunities to rebalance HSC states or interrupt state-specifying axes, limiting matrix-driven tumor promotion while sparing protective cytokine functions [9].

Multiomics now predicts therapy response by resolving stromal subtypes and their niches. In HCC, enrichment of POSTN^+^ CAFs identified by integrated single-cell/spatial analyses predicted diminished benefit from anti-programmed cell death protein 1 (PD-1)/PD-L1, nominating POSTN^+^ stroma as a resistance biomarker and target [28]. In colorectal cancer, spatial transcriptomics of the tumor–stroma boundary showed CXCL14^+^ CAF-rich, collagen-remodeling barriers in nonresponders, whereas LAMP3^+^ dendritic cells and CXCL13^+^ T-cell niches marked responders, linking stromal states to checkpoint blockade efficacy [29]. Complementarily, a tumor–immune barrier signature in HCC derived from multiomic profiling predicted immunotherapy responses, underscoring that stromal architecture captured by multiomics is clinically actionable [30]. The major stromal lineages/subtypes in the TME are concluded in Table 1.

**Table 1.** Major stromal lineages/subtypes in the TME: markers, spatial niches, functions, exemplar contexts, and key evidence. ↑ denotes “expression up-regulation” (gene/protein levels significantly higher than those in other TME stromal cells or normal counterparts), while ↓ denotes “expression down-regulation” (gene/protein levels significantly lower than those in other TME stromal cells or normal counterparts).

Cell subtype	Representative markers	Typical spatial niche	Core functions/phenotypes	Tumor cite	Key references
myCAF (myofibroblastic)	ACTA2/α-SMA, TAGLN, COL1A1	Tumor borders, invasive fronts	ECM synthesis/contractility; matrix stiffening	PDAC (pancreas)	[31]
iCAF (inflammatory)	IL-6↑, CXCL12↑, PDGFRA	Interstitial stroma away from nests	JAK/STAT3 inflammatory circuits; cytokine/chemokine exchange with tumor cells	PDAC (pancreas)	[31]
apCAF (antigen-presenting)	MHC-II, CD74 (low costimulation)	Near tumor nests/TLS rims	Cognate but tolerogenic CD4^+^ T-cell interactions; mesothelial contribution	PDAC	[32]
vCAF (vascular-associated)	NOTCH3, NR2F2, EPAS1, COL18A1	Perivascular sleeves	NOTCH3-programmed stromal activation; collagen deposition; invasion	Lung adenocarcinoma	[33]
FAP^+^ CAFs (functional bucket)	FAP, CXCL12	Stromal rims and fibrotic zones	Immune exclusion; targetable for imaging/therapy	PDAC; GI; breast	[158]
ifnCAF (interferon-response CAF)	IDO, CXCL9, CXCL10, CXCL11, IL-32	Tumor–stroma interface; closest to tumor cells in IMC	IFN-response program; associated with inflamed TME and favorable prognosis	Breast; NSCLC	[17,33]
tCAF (tumor-like CAF)	PDPN, MME (CD10), ENO1, NDRG1, CA9 (including stress/hypoxia-like states)	Tumor–stroma interface; frequent tumor contact	Tumor growth/angiogenesis-like program; associated with poor prognosis	Breast; NSCLC	[17,33]
mCAF (matrix CAFs)	MMP-11, CDH11, POSTN, collagen genes	Throughout stroma	ECM/stromal maintenance; linked to immune-poor stroma and worse survival	Breast; NSCLC	[17,33]
POSTN^+^ CAF neighborhoods (HCC barrier-associated)	POSTN (often α-SMA^+^); IL-6 ligand activity; ECM/TGF-β programs	Spatial tumor immune barrier regions	T-cell exclusion; IL-6–STAT3 coupling to SPP1^+^ macrophages; reduced ICI benefit	HCC	[28]
CXCL14^+^ CAF-rich collagen-remodeling barrier (CRC boundary)	CXCL14 (often COL1A1^+^CXCL14^+^); boundary ECM program (including MMP-11 induction via IHH–PTCH1)	Tumor–stroma boundary (≈0 ± 150 μm)	Structural barrier in nonresponders; contrasts with immune-activating boundary (LAMP3^+^ DC/CXCL13 signaling)	CRC	[29]
S1-CAF (matrix/immune-suppressive rim)	COL1A1, PD-L1, TGFB1	Tumor–stroma interface; dense collagen rims	Physical + immunologic barrier; EMT priming	Multiple solids	[2]
S2-CAF (inflammatory neighborhood)	IL6↑	Stromal zones away from nests	STAT3-driven inflammatory loops	Pan-cancer	[2]
S3-CAF (perivascular)	PDGFR, MMP-2	Vessel-adjacent	Stromal–immune cross-talk; perivascular remodeling	Pan-cancer	[2]
S4-CAF (TLS-related)	HLA-II, CXCL9, CCL21	Tertiary lymphoid structures	Adaptive-immunity modulation	Pan-cancer	[2]
TAECs (proliferative/angiogenic)	VEGFR2/KDR, ANGPT2	Hypoxic/angiogenic fronts	Sprouting angiogenesis	Multiple solids	[39]
TAECs (quiescent/steady)	CD36, ESAM; FA-transport/junctional genes	Stable capillaries	Barrier and nutrient transport homeostasis	Multiple solids	[39]
TAECs (abnormal/leaky)	↓VE-cadherin (CDH5), ↓CLDN5	Peri-necrotic/EMT-active zones	Barrier failure; chaotic flow; intravasation	Multiple solids	[42]
Pericytes (contractile)	ACTA2, MYH11, TAGLN	Enwrapping microvessels	Flow regulation; vessel stability	Pan-cancer	[44]
Pericytes (noncontractile/proangiogenic)	↓ACTA2/MYH11; ↑ECM/GF (e.g., ANGPT axes)	Angiogenic fronts	Sprouting support at cost of stability	Pan-cancer	[45]
Myofibroblastic hepatic stellate cells (myHSCs)	ACTA2, COL1A1	HCC stroma	ECM production; tumor-promoting	HCC	[27]
Cytokine-expressing hepatic stellate cells (cyHSCs)	IL-10^+^/TGFB1^−^ (context-dependent)	HCC stroma	Cytokine-secreting, tumor-constraining program	HCC	[27]

TME, tumor microenvironment; CAF, cancer-associated fibroblast(s); myCAF, myofibroblastic cancer-associated fibroblast(s); iCAF, inflammatory cancer-associated fibroblast(s); apCAF, antigen-presenting cancer-associated fibroblast(s); vCAF, vascular-associated cancer-associated fibroblast(s); FAP, fibroblast activation protein; GI, gastrointestinal; ifnCAF, interferon-response cancer-associated fibroblast(s); IFN, interferon; IDO1 (IDO), indoleamine 2,3-dioxygenase 1; CXCL9, C-X-C motif chemokine ligand 9; CXCL10, C-X-C motif chemokine ligand 10; CXCL11, C-X-C motif chemokine ligand 11; IL-32, interleukin-32; IMC, imaging mass cytometry; NSCLC, non-small cell lung cancer; tCAF, tumor-like cancer-associated fibroblast(s); mCAF, matrix cancer-associated fibroblast(s); POSTN, periostin; HCC, hepatocellular carcinoma; ICI, immune checkpoint inhibitor(s); CRC, colorectal cancer; PDAC, pancreatic ductal adenocarcinoma; TNBC, triple-negative breast cancer; ACTA2, actin alpha 2 (smooth muscle); α-SMA, alpha-smooth muscle actin; TAGLN, transgelin; COL1A1, collagen type I alpha 1 chain; ECM, extracellular matrix; IL-6, interleukin-6; CXCL12, C-X-C motif chemokine ligand 12 (stromal cell-derived factor 1, SDF-1); SDF-1, stromal cell-derived factor 1; CXCR4, C-X-C chemokine receptor type 4; PDGFRA, platelet-derived growth factor receptor alpha; JAK, Janus kinase; STAT3, signal transducer and activator of transcription 3; MHC-II, major histocompatibility complex class II; CD74, cluster of differentiation 74 (invariant chain); TLS, tertiary lymphoid structure; CD4, cluster of differentiation 4; NOTCH3, neurogenic locus notch homolog 3; NR2F2, nuclear receptor subfamily 2 group F member 2 (COUP-TFII); EPAS1, endothelial PAS domain protein 1; HIF-2α, hypoxia-inducible factor 2 alpha; COL18A1, collagen type XVIII alpha 1 chain; PDPN, podoplanin; MME (CD10), membrane metallo-endopeptidase (cluster of differentiation 10); ENO1, enolase 1; NDRG1, N-myc downstream regulated gene 1; CA9, carbonic anhydrase 9; MMP-11, matrix metalloproteinase 11; CDH11, cadherin 11; TGF-β, transforming growth factor-beta; TGFB1, transforming growth factor-beta 1; SPP1, secreted phosphoprotein 1 (osteopontin); FOXP3, forkhead box P3; Treg, regulatory T cell(s); CXCL14, C-X-C motif chemokine ligand 14; IHH, Indian hedgehog; PTCH1, patched 1; LAMP3, lysosomal-associated membrane protein 3; DC, dendritic cell(s); CXCL13, C-X-C motif chemokine ligand 13; PD-L1, programmed death-ligand 1; EMT, epithelial–mesenchymal transition; S1-CAF, state-1 cancer-associated fibroblast (matrix/immune-suppressive rim); S2-CAF, state-2 cancer-associated fibroblast (inflammatory neighborhood); S3-CAF, state-3 cancer-associated fibroblast (perivascular); S4-CAF, state-4 cancer-associated fibroblast (tertiary lymphoid structure-related); PDGFR, platelet-derived growth factor receptor; MMP-2, matrix metalloproteinase 2; HLA-II, major histocompatibility complex class II (human leukocyte antigen class II); CCL21, C-C motif chemokine ligand 21; TAECs, tumor-associated endothelial cells; TAEC, tumor-associated endothelial cell; TEC, tumor endothelial cell; VEGFR2, vascular endothelial growth factor receptor 2; KDR, kinase insert domain receptor (VEGFR2); ANGPT2, angiopoietin 2; CD36, cluster of differentiation 36 (fatty-acid translocase); ESAM, endothelial cell-selective adhesion molecule; FA, fatty acid; VE-cadherin, vascular endothelial cadherin; CDH5, cadherin 5; CLDN5, claudin 5; MYH11, myosin heavy chain 11; ANGPT, angiopoietin; GF, growth factor; myHSCs, myofibroblastic hepatic stellate cells; cyHSCs, cytokine-expressing hepatic stellate cells; IL-10, interleukin-10

## Heterogeneity and Functional Roles of Major Stromal Cell Populations in TME

### Cancer-associated fibroblasts

**Fig. 2.**
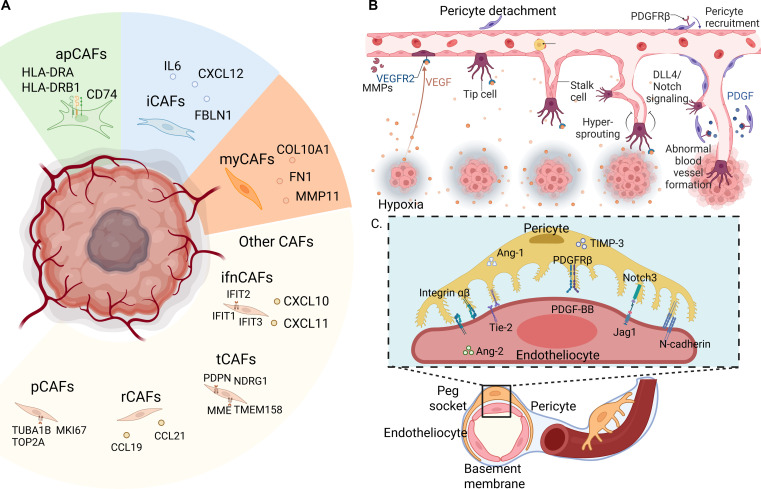
Stromal heterogeneity and spatial niches across the TME. (A) Heterogeneous cancer-associated fibroblast (CAF) subsets with distinct marker genes. (B) Pathological vascular formation involving pericyte detachment.(C) Physiological pericyte–endotheliocyte cross-talk in vascular homeostasis.

CAFs are abundant and highly plastic stromal cells that remodel ECM, modulate inflammatory cues, and influence antitumor immunity across solid tumors. Single-cell and spatial multiomics analyses have converged on 4 conserved CAF archetypes with distinct transcriptional programs and spatial niches, as illustrated by the diverse CAF subsets (e.g., myCAFs, iCAFs, apCAFs, tCAFs, ifnCAFs, pCAFs, and rCAFs) and their signature markers in Fig. 2A. Myofibroblastic CAFs (myCAFs) express ACTA2 and TAGLN, concentrate at tumor–stromal interfaces, and specialize in matrix deposition and contractility, while iCAFs secrete cytokines such as IL-6 and chemokines like CXCL12 and can interconvert with myCAFs through opposing IL-1–Janus kinase/signal transducer and activator of transcription (JAK/STAT) and TGF-β–SMAD signaling [31]. Antigen-presenting CAFs (apCAFs) up-regulate MHC-II and CD74 yet lack classical costimulation, enabling cognate interactions with CD4^+^ T cells that tend to be tolerogenic, and lineage tracing indicates that mesothelial cells can give rise to apCAFs during pancreatic tumor progression [32]. Vascular-associated CAFs (vCAFs) are defined by markers including MCAM, NOTCH3, and COL18A1; reside near microvessels; and exemplify perivascular cross-talk through NOTCH3-programmed stromal activation that enhances collagen production and invasion in lung adenocarcinoma [33]. Large pan-cancer spatial atlases integrating transcriptomic and proteomic platforms validate these 4 CAF states as conserved and spatially organized neighborhoods, linking subtype composition to immune contexture and clinical outcomes. Collectively, this framework supports subtype-aware therapeutic strategies that target TGF-β/ECM programs or disrupt pathogenic NOTCH signaling in selected contexts [34].

### Vascular endothelial cells

Tumor-associated endothelial cells (TAECs) within the TME display marked functional and molecular heterogeneity beyond their conventional role in the blood circulation, as shown in Fig. 2B [35]. Single-cell transcriptomics has identified several distinct TAEC subpopulations: proliferative TAECs, which express VEGFR2 and angiopoietin-2 and localize to angiogenic regions such as the invasive front and hypoxic niches [36]. A quiescent state is enriched in vessels with stable junctions and higher expression of fatty-acid transport and junctional programs, exemplified by CD36 in capillary endothelium and tight-junction molecules such as ESAM in homeostatic vessels [37]. An abnormal state shows reduced VE-cadherin and claudin-5 with barrier failure, driving leakiness that facilitates intravasation, extravasation, and disordered flow [38]. TAECs also perform diverse functions including the formation of the blood–tumor barrier, which exhibits altered permeability affecting drug delivery and immune infiltration [39]. They also rewire nutrient trafficking to buffer metabolic stress and support tumor bioenergetics [40]. In metastatic seeding, endothelial E-selectin promotes arrest and adhesion of circulating tumor cells, and angiocrine signals from endothelium foster colonization competence [41]. Spatially, TAEC composition tracks progression, with proliferative TAECs increased in advanced or hypoxic regions of solid tumors [36]. Abnormal TAECs frequently align with perinecrotic zones or epithelial–mesenchymal transition (EMT)-active territories in which leakiness and angiogenic remodeling colocalize [42]. This evolving heterogeneity highlights stage-aware vascular strategies that pair normalization or anti-angiogenic agents with immunotherapy or metabolism-targeted approaches.

### Pericytes

Recent multiomics technologies have uncovered significant heterogeneity in pericytes within the TME [43]. Two main subtypes have been identified, as contractile pericytes, which express high levels of α-SMA/ACTA2 and SM-MHC/MYH11, enwrap microvessels, and regulate blood flow through contraction [44], and noncontractile pericytes, which exhibit reduced contractility but produce ECM components and growth factors and often localize to angiogenic fronts to support neovessel formation [45]. Signaling pathways such as TGF-β, Notch, and Wnt regulate transitions toward a less-contractile, proangiogenic phenotype that promotes vessel growth at the expense of vascular stability [46].

Pericytes maintain intimate cross-talk with endothelial cells via key molecular pathways, as detailed in the molecular interactions between pericytes and endothelial cells in Fig. 2C. The PDGF-BB/PDGFR-β axis mediates pericyte recruitment and vessel coverage, while pericyte-derived angiopoietin-1 activates endothelial Tie2 receptors to enhance junctional integrity [47]. Through gap junctions and paracrine signals including TGF-β and vascular endothelial growth factor (VEGF), pericytes further modulate vascular permeability and stability, and dysregulation of these interactions in tumors contributes to abnormal vessel function and leakage [48].

Beyond structural roles, pericytes actively facilitate metastasis. Detachment from vessels, driven by protease activity, signaling imbalances, or mechanical stress, creates gaps that promote tumor cell intravasation and extravasation [49]. Detached pericytes can transition into CAFs and secrete proinvasive mediators such as TGF-β and CCL2 that foster EMT and invasion [50]. Moreover, pericytes contribute to premetastatic niche formation via matrix remodeling and cytokine secretion [51]. Low pericyte coverage correlates with poor prognosis and heightened metastatic risk in several settings, underscoring their role as active mediators of cancer dissemination rather than mere structural supporters [52].

### Tumor-type-specific stromal ecosystems

While pan-cancer single-cell/spatial atlases support a conserved stromal backbone, the relative abundance, spatial deployment, and immune coupling of stromal programs vary markedly by tumor type, creating tissue-specific stromal ecosystems with distinct therapeutic implications. In PDAC, desmoplasia is dominated by spatially segregated fibroblast programs, including tumor-interface myCAFs and more interstitial iCAFs, which can interconvert under opposing IL-1–JAK/STAT versus TGF-β–SMAD cues. PDAC also features apCAFs enriched near tumor/TLS-like rims, supporting tolerogenic CD4^+^ T-cell interactions [31,32]. In HCC, stromal architecture is strongly shaped by HSC state continua, including matrix-producing myofibroblastic HSC (myHSC) programs versus cytokine-expressing, potentially tumor-constraining states, emphasizing that CAF-like activation in liver cancers has a lineage-specific backbone [27]. Multiomics further links HCC stroma to immunotherapy outcomes: enrichment of POSTN^+^ CAF neighborhoods and tumor immune barrier features correlates with reduced benefit from anti-PD-1/PD-L1, nominating specific stromal states as predictive resistance biomarkers and targets [28,30]. In breast cancer, spatial profiling reveals pronounced plasticity of immunosuppressive fibroblast neighborhoods and their alignment with immune exclusion patterns, reinforcing that fibroblast-mediated suppression can be organized into discrete spatial programs rather than diffuse activation [53]. Finally, in CRC, spatially organized tumor–stroma boundaries can stratify immunotherapy response, where CXCL14^+^ CAF-rich collagen-remodeling barriers are enriched in nonresponders, whereas responder regions show immune-activating niches (e.g., LAMP3^+^ DC and CXCL13^+^ T-cell neighborhoods), directly connecting boundary stromal states to clinical efficacy [29].

**Fig. 3.**
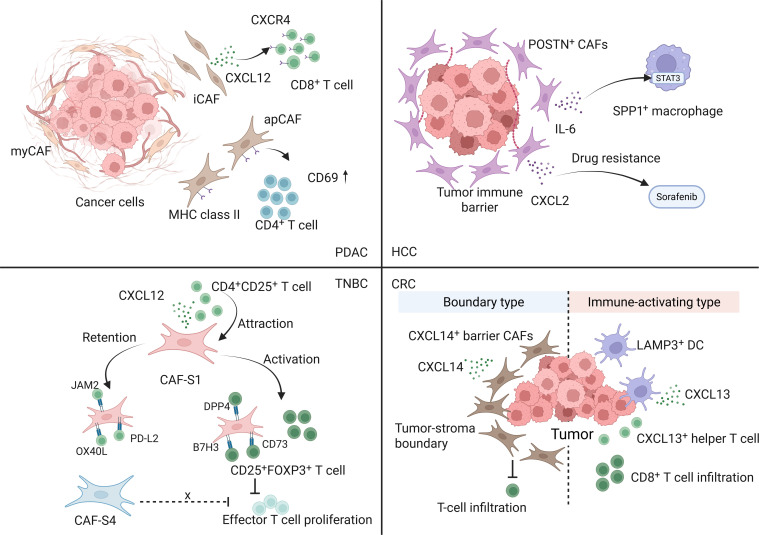
Tumor-type-specific stromal–immune circuits shaping immune exclusion and therapeutic response. Schematic overview of representative stromal niches across tumor types. PDAC: myCAF/iCAF programs contribute to barrier formation; iCAFs signal via CXCL12–CXCR4 to promote T-cell exclusion; apCAFs express MHC-II and engage CD4^+^ T cells. HCC: POSTN^+^ CAFs form a spatial immune barrier and activate an IL-6–STAT3 axis in SPP1^+^ macrophages, linking myeloid immunosuppression to therapy resistance. TNBC: CAF-S1 attracts/retains CD4^+^CD25^+^ T cells via CXCL12 and promotes FOXP3^+^ Treg-skewing through additional ligand–receptor interactions. CRC: tumor–stroma boundary states stratify ICI outcomes, with CXCL14^+^ barrier CAFs limiting infiltration versus an immune-activating boundary enriched for LAMP3^+^ DC/CXCL13 signaling that supports T-cell entry. PDAC, pancreatic ductal adenocarcinoma; HCC, hepatocellular carcinoma; TNBC, triple-negative breast cancer; CRC, colorectal cancer; CAF, cancer-associated fibroblast(s); myCAF, myofibroblastic cancer-associated fibroblast(s); iCAF, inflammatory cancer-associated fibroblast(s); apCAF, antigen-presenting cancer-associated fibroblast(s); CXCL12, C-X-C motif chemokine ligand 12 (stromal cell-derived factor 1, SDF-1); CXCR4, C-X-C chemokine receptor type 4; MHC-II, major histocompatibility complex class II; CD4^+^ T cell, cluster of differentiation 4-positive T cell; CD25^+^ T cell, cluster of differentiation 25-positive T cell (interleukin-2 receptor alpha-positive T cell); POSTN, periostin; IL-6, interleukin-6; STAT3, signal transducer and activator of transcription 3; SPP1, secreted phosphoprotein 1 (osteopontin); FOXP3, forkhead box P3; Treg, regulatory T cell(s); ICI, immune checkpoint inhibitor(s); CXCL14, C-X-C motif chemokine ligand 14; LAMP3, lysosomal-associated membrane protein 3; DC, dendritic cell(s); CXCL13, C-X-C motif chemokine ligand 13.

These tumor-type differences imply that stromal interventions should be tumor-specific and niche-aware rather than uniformly CAF-targeted. In PDAC, barrier-like myCAF-rich rims and cytokine/chemokine circuits from iCAFs/apCAFs plausibly contribute to immune exclusion and poor drug penetration, suggesting that combination strategies may need to match the dominant local program (ECM/TGF-β-skewed versus inflammatory IL-6/CXCL12-skewed) identified by single-cell/spatial readouts [31,32]. In HCC, multiomics-defined barrier states (e.g., POSTN^+^ CAF enrichment and immune-barrier signatures) provide a rationale for patient stratification and for pairing checkpoint blockade with stromal reprogramming aimed at dismantling barrier-like niches [28,30]. In breast cancer/TNBC, treatment-induced remodeling of stromal–immune circuits can follow distinct response trajectories under immunotherapy-based regimens, emphasizing that stromal states are dynamic and may require on-treatment monitoring rather than one-time pretreatment labeling [54]. In CRC, the spatial logic of the tumor–stroma boundary offers a practical framework to interpret why otherwise similar tumor-intrinsic features yield different immunotherapy outcomes, and motivates boundary-targeted stromal normalization to unlock immune infiltration [29]. Collectively, these findings justify adding a tumor-type-resolved stromal ecosystem layer on top of conserved subtype taxonomies, and we summarize the corresponding stromal–immune cross-talk in a single-cell-resolved schematic (Fig. 3).

## Mechanisms of Stromal Cell-Derived Cytokines in Regulating Tumor Metastasis

### Key cytokine signaling axes mediating CAF-tumor cell cross-talk

Cytokine-mediated communication between CAFs and tumor cells is a fundamental driver of metastatic progression, orchestrating processes from local invasion to distant colonization [50]. The TGF-β pathway serves as a central regulator in this stromal–tumor cross-talk, integrating canonical SMAD signaling with noncanonical cascades [55]. CAF-derived TGF-β activates SMAD-dependent and MAPK/phosphoinositide 3-kinase (PI3K)–protein kinase B (AKT) signaling in tumor cells, inducing EMT through E-cadherin loss and up-regulation of N-cadherin, vimentin, and EMT-TFs such as Snail and Twist [56]. Concurrently, TGF-β programs CAFs to enhance matrix remodeling by up-regulating MMP-2 and MMP-9 that facilitate ECM degradation and promotes collagen cross-linking via lysyl-oxidase family enzymes, yielding a stiffened matrix that supports invasion through mechanosensing [57].

IL-6 signaling in the CAF–tumor axis centers on the IL-6R/gp130 complex, which robustly activates JAK/signal transducer and activator of transcription 3 (STAT3) and also engages the PI3K/AKT pathway. AKT, in turn, functionally inhibits GSK3β, converging on prostemness and prosurvival programs [58,59]. In cancer cells, CAF-derived IL-6 phosphorylates STAT3 and up-regulates stemness drivers (NANOG, OCT4, and SOX2), a phenotype linked to therapy resistance [60,61]. STAT3 also promotes apoptosis evasion by transcriptionally inducing anti-apoptotic effectors such as BCL-2 and MCL-1 [62,63]. Moreover, STAT3 cooperates with NF-κB and HIF-1α to create a self-reinforcing inflammatory–hypoxic loop that sustains metastatic signaling [64,65].

At the vascular interface, IL-6 trans-signaling (via sIL-6R) activates STAT3/AKT/ERK in endothelial cells and up-regulates adhesion/coagulation cues linking local tumor cytokine flux to vascular effects [66,67]. Systemically, classic IL-6 signaling in hepatocytes is a principal driver of the acute-phase response (e.g., C-reactive protein and fibrinogen), thereby connecting paracrine tumor IL-6 to hepatic and circulatory consequences; notably, sgp130-Fc selectively blocks IL-6 trans-signaling [68].

Metastatic dissemination is reinforced by a layered network of cytokine and growth-factor signals that operate in parallel and with substantial redundancy. The CXCL12–CXCR4 axis directs tumor-cell homing to permissive niches such as bone marrow [69,70], and hepatocyte growth factor (HGF)–MET signaling promotes collective migration and survival via the adaptor GAB1, activating downstream PI3K–AKT/MAPK pathways [71]. Functionally, these partially overlapping circuits safeguard metastatic competence even under therapeutic pressure by preserving motility, survival, and niche adaptation.

Within desmoplastic PDAC, CAF-derived leukemia inhibitory factor–leukemia inhibitory factor receptor signaling augments chemoresistance and may be leveraged alongside cytotoxic backbones [72]. In the same stressed stromal ecosystems, IL-11 acting through IL-11Rα/gp130–STAT3 further reinforces anti-apoptotic programs and EMT [73]. Inflammatory chemokine circuits add yet another layer: CXCL8/interleukin-8 (IL-8) engagement of CXCR1/2 maintains invasive, stem-like states and is the focus of active clinical efforts [74], while CCL5–CCR5 signaling promotes migration, invasion, and prometastatic conditioning across tumor types [75].

In parallel, receptor tyrosine kinase (RTK) programs provide fail-safe redundancy. GAS6–AXL supports EMT, invasion, and survival within hypoxic, invasive niches. Consistent with this theme, HGF–MET drives collective migration and prosurvival signaling via a GAB1-scaffolded PI3K–AKT/MAPK cascade. Conceptually, RTK coactivation broadens signaling bandwidth and guards against single-node therapeutic blockade [76].

Guidance cues further diversify the prometastatic signaling repertoire. In HCC, SEMA3C signaling through NRP1/ITGB1 establishes a bidirectional tumor–stellate/CAF loop that amplifies NF-κB/IL-6 programs and fosters therapy resistance [4]. SEMA4D–PLXNB1 can engage PI3K–AKT and cross-talk with c-MET in aggressive contexts [77], whereas SLIT2–ROBO1 shows tissue-specific duality, promoting PDAC liver outgrowth but restraining tumor programs in other settings [78]. These guidance pathways integrate positional information with stromal feedback, effectively tuning metastatic behavior to organ context.

At the tumor–stroma interface, CAF-derived morphogens orchestrate vascular, immune, and invasive phenotypes. WNT2 and WNT5A signaling through FZD and ROR receptors influences angiogenesis, immune evasion, and invasion [79]. Contact-dependent JAG1–NOTCH signaling at tumor–stroma interfaces entrenches EMT, stemness, and context-dependent therapeutic resistance [80]. Additional growth-factor streams, including PDGF and FGF2 acting through FGFRs, drive proliferation, migration, and stromal remodeling in perivascular and fibrotic zones [81,82]. Extracellular-matrix-encoded signals such as osteopontin (OPN/SPP1) via CD44/integrins and periostin (POSTN) via αv-integrins promote stemness and metastatic competence within dense desmoplasia [83].

Epigenetic regulation is increasingly recognized as a key layer that couples microenvironmental cues to durable yet reversible stromal cell states, thereby enabling CAF plasticity and subtype switching. In PDAC, tumor cells can actively reprogram fibroblasts through induced DNA methylation programs, exemplified by methylation-dependent suppression of negative regulators (e.g., SOCS1) and downstream activation of protumor signaling such as STAT3, ultimately promoting tumor growth [84]. Complementarily, metabolic cues can directly reshape the CAF epigenome: tumor-derived lactate flux activates TET-dependent demethylation, leading to widespread loss of cytosine methylation and transcriptional rewiring during CAF formation [85]. Beyond DNA methylation, histone modifications provide an epigenetic memory that stabilizes tumor-promoting programs. In breast stroma, cancer cell exposure reduces EZH2 occupancy and H3K27 trimethylation at specific promoters (e.g., ADAMTS1), producing a heritable invasive CAF-like phenotype [86]. Consistently, loss of H3K27me3 in CAFs is enriched at genes encoding stem-cell niche and stromal–epithelial interaction factors and functionally associates with enhanced tumor-promoting capacity [87]. Importantly, chromatin readers/writers/erasers can be therapeutically leveraged to reprogram stroma: BET bromodomain inhibition (JQ1) disrupts TGF-β-dependent transcriptional machinery in CAFs and suppresses CAF activation programs in vivo, whereas histone deacetylase inhibition can paradoxically create a permissive chromatin landscape that unleashes AP-1-driven inflammatory (SASP-like) cytokine programs in CAFs, highlighting the need for rational combinations [88]. Finally, single-cell chromatin accessibility analyses provide direct evidence for state transitions (e.g., pre-CAF to CAF) and enhancer–gene links that underlie stromal lineage plasticity during malignant progression [89].

Finally, matrix remodeling consolidates the premetastatic state at the biophysical level. Lysyl oxidases (LOX/LOXL) stiffen and hypoperfuse the stroma while priming distant niches; pharmacologic pan-LOX inhibition (PXS-5505) can normalize mechanics and improve drug delivery in PDAC models [90,91]. This convergence of cytokine, RTK, guidance, morphogen, and matrix programs argue for rational, multiaxis therapeutic strategies that disrupt both biochemical signaling and biomechanical conditioning, with the aim of eroding metastatic readiness despite ongoing treatment pressure. The detailed information is shown in Table 2.

**Table 2.** “Stroma → tumor” cytokine and guidance axes

Axis (class)	Stromal sources → Tumor targets (receptors)	Canonical downstream	Core biological effects	Spatial/systemic features	Therapeutic notes	Key references
TGF-β (morphogen)	CAFs → tumor and CAFs (TβRI/II)	SMAD2/3/4; PI3K/AKT; MAPK	EMT, invasion, fibroblast activation; MMPs; collagen cross-talk	Steep gradients at stromal rims; latent ECM-tethered pools	Localized/ECM-aware delivery; combo with IO/chemo	[55]
IL-6 (cytokine)	CAFs ↔ tumor (IL-6R/gp130)	JAK/STAT3; PI3K/AKT; GSK3β	Stemness, survival, acute-phase cues; EC activation	Tumor paracrine and systemic hepatic/vascular effects	IL-6/IL-6R blockade; rational IO/chemo combinations	[58,59]
CXCL12/SDF-1 → CXCR4 (chemokine)	FAP^+^ CAFs and stroma → CXCR4^+^ tumor/immune cells	PI3K/AKT; MAPK	Chemotaxis; immune exclusion; organotropic metastasis	Directed gradients to vessels, BM/LN niches	Plerixafor/other CXCR4 antagonists; timing to preserve hematopoiesis	[69,70]
HGF → MET (growth factor)	CAFs/stroma → MET^+^ tumor	RAS/MAPK; PI3K/AKT; STAT3 via GAB1	Collective migration, survival, drug resistance	Enriched at invasive/vascular fronts	MET inhibitors; consider combos with IO/ECM normalization	[71]
LIF → LIFR (cytokine)	CAFs → tumor	JAK/STAT3	Stemness, EMT state, chemoresistance	PDAC desmoplasia; stromal loops	LIF/LIFR inhibitors with chemotherapy	[72]
IL-11 → IL-11Rα (cytokine)	CAFs (including therapy-stressed) → tumor	JAK/STAT3	Anti-apoptosis (↑BCL-2/Survivin); EMT; drug resistance	Induced by chemotherapy in CAFs	IL-11/STAT3 targeting to reverse resistance	[73]
CXCL8/IL-8 → CXCR1/2 (chemokine)	CAFs → tumor	SRC→EGFR/HER2 transactivation; ERK	Invasion, stemness; therapy resistance	Often at inflammatory rims	Anti-CXCL8 mAbs/CXCR1/2 antagonists in trials	[74]
CCL5 → CCR5 (chemokine)	CAFs/stroma → CCR5^+^ tumor	NF-κB; PI3K; RHO	Migration/invasion; DNA-damage response; stemness	High in metastatic niches	CCR5 antagonists (repurposing) under study	[75]
GAS6 → AXL (TAM ligand/RTK)	CAFs/mesenchyme → AXL^+^ tumor	PI3K/AKT; MAPK; NF-κB	EMT, invasion, survival	Hypoxic/invasive fronts	AXL inhibitors (e.g., bemcentinib) combinations	[76]
SEMA3C → NRP1/ITGB1 (axon guidance)	HCC cells↔HSCs/CAFs (loop)	NF-κB; ↑IL-6; ↑cholesterol biosynthesis	HSC activation; matrix contraction; therapy resistance	HCC stroma; bidirectional feed-forward	Block SEMA3C/NRP1 or upstream TGF-β	[4]
SEMA4D → PLXNB1 (axon guidance)	Stromal/immune (including CAF/TAM/osteoclast) → tumor	RHOA/ROCK; PYK2/SRC; c-MET cross-talk	Invasion; angiogenesis; perineural/osseous remodeling	At invasive and bone niches	Anti-SEMA4D antibodies explored with IO	[77]
SLIT2 → ROBO1 (axon guidance)	CAFs → ROBO1^+^ tumor	PI3K/AKT/β-catenin (context-dependent)	Context-specific: tumor-suppressive in several settings; prometastatic in others	Often stromal secretion at tumor edge	Axis is bidirectional; consider context before targeting	[78]
WNT2/WNT5A → FZD/ROR (morphogens)	CAFs → tumor	β-catenin-dependent and noncanonical WNT	Invasion, migration; immune modulation	Peri-tumoral stroma; angiogenic zones	WNT-axis inhibitors; consider toxicity	[79]
JAG1 (Notch ligand) → NOTCH (cell–cell)	CAFs → tumor	NOTCH→HES/HEY	EMT/stemness; therapy resistance (context-dependent)	Hypoxic/invasive niches; direct contact	γ-secretase/Notch-ligand strategies; careful patient selection	[80]
PDGF (growth factor)	CAFs/pericytes → PDGFR^+^ tumor	PI3K/AKT; MAPK	Proliferation, migration; stromal remodeling	Perivascular and fibrotic zones	Multitarget TKIs (context dependent)	[81]
FGF2 → FGFR (growth factor)	CAFs → FGFR^+^ tumor	RAS/MAPK; PI3K/AKT	Proliferation; EMT; resistance	Stromal hubs under stress	FGFR inhibitors (tumor-genotype guided)	[82]
OPN/SPP1 → CD44/integrins (ECM cue)	CAFs/stroma → tumor	FAK/SRC; MAPK; NF-κB	Stemness; migration; immune modulation	Fibrotic rims	Anti-integrin/FAK strategies; biomarker role	[83]
Periostin (POSTN) → αv integrins (ECM cue)	CAFs (PDPN^+^) → tumor	FAK→YAP/TAZ	Stemness and metastasis programs	Dense desmoplastic stroma	FAK/YAP pathway targeting	[199]
LOX/LOXL (ECM enzyme)	CAFs/hypoxic tumor/ECs → ECM (collagen/elastin)	Cross-linking enzymes	↑Stiffness/solid stress; perfusion failure; PMN priming	Hypoxic rims; distant premetastatic organs	Pan-LOX inhibition (e.g., PXS-5505) with chemo improves perfusion in PDAC models	[90,91]

TGF-β, transforming growth factor-beta; CAF, cancer-associated fibroblast; TβRI/II, transforming growth factor-beta receptor type I/II; SMAD2/3/4, SMAD family member 2/3/4; PI3K, phosphoinositide 3-kinase; AKT, protein kinase B; MAPK, mitogen-activated protein kinase; EMT, epithelial–mesenchymal transition; MMPs, matrix metalloproteinases; ECM, extracellular matrix; IO, immuno-oncology; IL-6, interleukin-6; IL-6R, interleukin-6 receptor; gp130, glycoprotein 130 (IL6ST); JAK, Janus kinase; STAT3, signal transducer and activator of transcription 3; GSK3β, glycogen synthase kinase-3 beta; EC, endothelial cell; CXCL12, C-X-C motif chemokine ligand 12; SDF-1, stromal cell-derived factor-1; CXCR4, C-X-C chemokine receptor 4; BM, bone marrow; LN, lymph node; FAP, fibroblast activation protein; HGF, hepatocyte growth factor; MET (c-MET), MET proto-oncogene receptor tyrosine kinase; RAS, rat sarcoma (RAS) GTPase; GAB1, GRB2-associated-binding protein 1; LIF, leukemia inhibitory factor; LIFR, leukemia inhibitory factor receptor; IL-11, interleukin-11; IL-11Rα, interleukin-11 receptor alpha; CXCL8 /IL-8, C-X-C motif chemokine ligand 8/interleukin-8; CXCR1, C-X-C chemokine receptor 1; CXCR2, C-X-C chemokine receptor 2; EGFR, epidermal growth factor receptor; HER2, human epidermal growth factor receptor 2 (ERBB2); ERK, extracellular signal-regulated kinase; CCL5, C-C motif chemokine ligand 5; CCR5, C-C chemokine receptor 5; NF-κB, nuclear factor kappa-B; RHO, Ras homolog family (Rho) GTPase; GAS6, growth arrest-specific protein 6; AXL, AXL receptor tyrosine kinase; TAM (receptors), TYRO3–AXL–MERTK receptor tyrosine kinase family; RTK, receptor tyrosine kinase; SEMA3C, semaphorin-3C; NRP1, neuropilin-1; ITGB1, integrin β1; HCC, hepatocellular carcinoma; HSCs, hepatic stellate cells; SEMA4D, semaphorin-4D; PLXNB1, plexin-B1; RHOA, Ras homolog family member A; ROCK, Rho-associated protein kinase; PYK2, proline-rich tyrosine kinase 2 (PTK2B); SRC, proto-oncogene tyrosine-protein kinase Src; SLIT2, slit guidance ligand 2; ROBO1, roundabout guidance receptor 1; β-catenin, beta-catenin; WNT2, Wnt family member 2; WNT5A, Wnt family member 5A; FZD, Frizzled class receptor; ROR, receptor tyrosine kinase-like orphan receptor; CRC, colorectal cancer; GC, gastric cancer; JAG1, Jagged-1; NOTCH, Notch receptor family; HES, Hairy and Enhancer of Split; HEY, Hes-related with YRPW motif; PDGF, platelet-derived growth factor; PDGFR, platelet-derived growth factor receptor; TKIs, tyrosine kinase inhibitors; FGF2, fibroblast growth factor 2; FGFR, fibroblast growth factor receptor; OPN, osteopontin; SPP1, secreted phosphoprotein-1 (gene for osteopontin); CD44, cluster of differentiation 44; FAK, focal adhesion kinase (PTK2); POSTN, periostin; PDPN, podoplanin; αv integrins, integrins containing the alpha-V subunit; YAP, Yes-associated protein; TAZ, transcriptional coactivator with PDZ-binding motif (WWTR1); LOX, lysyl oxidase; LOXL, lysyl oxidase-like protein; PMN, premetastatic niche; PXS-5505, pan-lysyl oxidase inhibitor; PDAC, pancreatic ductal adenocarcinoma

### Spatial specificity of cytokine action

Stromal-derived cytokines’ spatial distribution (via localized production, ECM sequestration, and concentration gradients) shapes the TME and metastatic progression [92]. Transforming growth factor-beta (TGF-β) concentrates at peritumoral rims and invasive fronts, where spatial profiling shows heightened TGF-β signaling and EMT programs relative to tumor cores, consistent with a peripherally polarized niche [93]. This polarization reflects stromal overproduction together with activation of latent TGF-β by proteases such as MMP-2 and MMP-9 and by αv-integrins that are enriched in invasion zones [94]. Latent TGF-β is tethered to ECM via LTBPs, creating a readily activatable reservoir that sustains gradients; interfering with matrix release of TGF-β confirms this reservoir function in situ [94]. Resultant TGF-β gradients drive EMT at the tumor–stroma interface and recruit or activate fibroblasts, amplifying a self-reinforcing TGF-β loop [95].

Interleukin-6 (IL-6) acts both locally and systemically [96]. Within primary tumors, it forms autocrine and paracrine loops between CAFs and tumor cells, sustaining STAT3 activation and stemness [61]. Systemically, IL-6 induces hepatic acute-phase proteins, such as serum amyloid A that prime premetastatic niches, and promotes prometastatic endothelial activation including adhesion-molecule up-regulation that facilitates tumor-cell arrest and transmigration [97]. IL-6 also intersects with bone remodeling by stimulating osteoclastogenic pathways through RANKL-dependent cues in the marrow ecosystem, thereby fostering skeletal colonization [98].

**Fig. 4.**
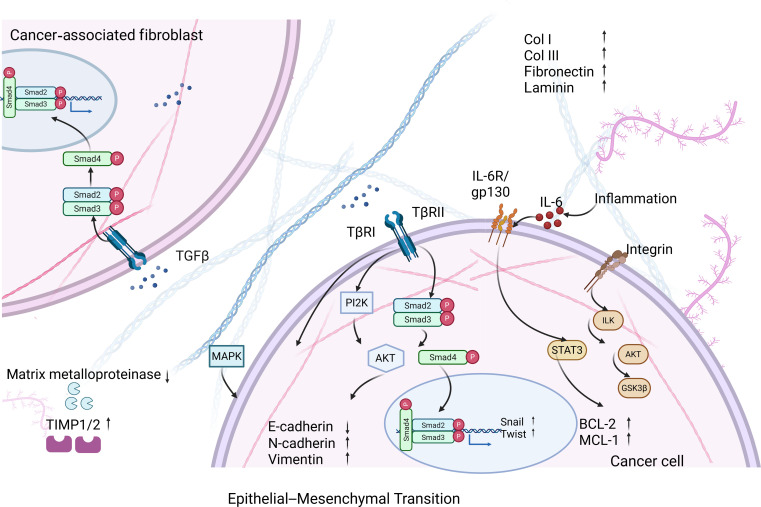
Cytokine circuits and spatial gradients driving metastasis. Key signaling pathways (TGF-β/SMAD, PI3K/AKT, MAPK, IL-6/STAT3) driven by CAFs activate a prometastatic program in cancer cells. This leads to EMT induction, increased cell survival, and ECM remodeling, collectively facilitating metastasis.

CXCL12 creates gradients guiding tumor cell migration. CAFs direct primary tumor cells toward blood vessels (aiding intravasation), while CXCR4^+^ tumor cells home to CXCL12-producing organs (bone marrow: perivascular stromal cells/osteoblasts; lymph nodes: fibroblastic reticular cells), explaining organotropic metastasis [99]. Figure 4 visually encapsulates the molecular cascades underlying these events:

These spatial dynamics have therapeutic implications [100]. Localized TGF-β blockade using tumor-targeted nanovesicles or ECM-aware delivery can intensify effects at invasive fronts while mitigating systemic toxicity [101]. IL-6 inhibition generally requires systemic administration but must be balanced against infection risk and potential interference with antitumor immunity [102]. Targeting the CXCL12/CXCR4 axis benefits from temporal and spatial control because this pathway is indispensable for hematopoietic trafficking and broad chemokine homeostasis [103].

### Multiomics evidence for cytokine-driven metastasis

Multiomics technologies have profoundly advanced our understanding of cytokine-driven metastasis, revealing specialized CAF subtypes that orchestrate prometastatic niches. Large pan-cancer spatial atlases now define conserved CAF programs whose abundance and neighborhoods correlate with immune state, therapy response, and survival [2]. Transcriptomic, proteomic, and spatial analyses consistently identify metastasis-promoting CAF signatures associated with poor clinical outcomes and therapeutic resistance. In melanoma and squamous cell carcinoma, spatial single-cell profiling resolves myofibroblastic, matrix, and immunomodulatory CAF states that expand with malignancy [104]. For pancreas, single-cell studies show functional CAF heterogeneity with myofibroblastic subsets that accelerate metastasis, while IL-1–IL1RAP signaling in CAFs drives chemokine output and neutrophil recruitment, directly tying inflammatory CAF circuits to dissemination [22]. Inflammatory axes extend to SAA family genes induced by IL-1 in CAFs, further linking stromal cytokines to neutrophil trafficking, and vascular-mimicry programs involve VE-cadherin (CD144)–β-catenin/TCF signaling that facilitates tumor channel formation and metastatic spread [105].

Functionally, these CAFs promote metastasis through extracellular-matrix remodeling and stemness induction (for example, via hyaluronan-rich niches that engage CD44), and by activating canonical cytokine pathways including TGF-β-induced EMT, JAK/STAT3, and CXCL12/CXCR4 chemotaxis, while recruiting neutrophils to premetastatic niches [106].

Clinically, CAF-derived gene signatures predict metastatic risk and poor survival; recent models in colorectal cancer and oral squamous cell carcinoma demonstrate that CAF-weighted panels or matrix-CAF markers stratify prognosis and align with aggressive disease biology [107].

## Clinical Translation of Stromal Cell-Targeted Therapies

### Targeting CAFs

Targeting CAFs has become a promising strategy, especially in stromal-rich cancers like PDAC; 2 major approaches are FAP-directed therapies and inhibition of the TGF-β pathway to disrupt protumorigenic CAF functions and recondition the immunosuppressive TME [108].

FAP, overexpressed on CAFs in many epithelial cancers, is being pursued with multiple modalities including antibody–drug conjugates (ADCs); recent platforms use exatecan-class topoisomerase-I payloads and FAP-targeted conjugates or SMDCs to debulk FAP^+^ stroma and enable immune engagement, and FAP-targeted radioligand/small-molecule drug conjugate approaches synergize with anti-PD-(L)1 to remodel the TME (increase CD8^+^/M1 and reduce myeloid-derived suppressor cells [MDSCs]/Tregs) and boost efficacy in preclinical models [109]. FAP-specific cellular therapies are also in early clinical exploration, with reviews summarizing ongoing FAP-CAR (chimeric antigen receptor) programs and broader CAR-T (chimeric antigen receptor T-cell therapy) progress in solid tumors [110]. In parallel, TGF-β inhibition seeks to mitigate CAF-mediated immunosuppression and metastasis. The antisense agent trabedersen (OT-101) targets TGF-β2 and has re-entered clinical testing in combination with pembrolizumab, while contemporary reviews and trials of small-molecule/antibody inhibitors (e.g., vactosertib; discontinued NIS793 program) frame the evolving landscape [111]. Preclinical and early-phase studies indicate that combining TGF-β blockade with cytotoxics can reduce fibroblast activation and tumor burden, consistent with stroma modulation that may improve treatment responses in PDAC [112]. Innovative bifunctional agents that simultaneously neutralize PD-L1 and TGF-β (e.g., SHR-1701, YM101, and the PD-L1/TGF-β bispecific Y101D now in phase 1) enhance T-cell infiltration and M1 polarization in models and have shown early clinical activity, including signals of efficacy in gastrointestinal and pancreatic cancer settings [113].

### Anti-angiogenic therapies

CAFs play a key role in therapeutic resistance by up-regulating alternative proangiogenic factors such as fibroblast growth factor (FGF), PDGF, IL-8, and angiopoietins following VEGF inhibition, and by sustaining angiogenesis through cytokine-driven programs [47]. Hypoxia-driven HIF-1α activation further promotes this compensatory angiogenesis, while ECM remodeling by CAFs reduces drug delivery and supports endothelial survival [114].

Resistance also involves the selection of abnormal vascular strategies with reduced VEGF dependence (for example, vessel co-option) and the recruitment of proangiogenic bone-marrow-derived myeloid cells [115]. These adaptations underscore the necessity for combination strategies [116]. A promising approach combines anti-angiogenic agents with immune checkpoint inhibitors (ICIs) [117]. VEGF inhibition can induce a transient “vascular normalization” window that improves perfusion, reduces hypoxia, and enhances drug penetration and immune-cell infiltration [118]. It also alleviates VEGF-mediated immunosuppression, supporting T-cell function and dendritic-cell maturation [119].

Clinically, the bevacizumab–atezolizumab combination in HCC (IMbrave150) improved overall survival and has emerging translational evidence linking benefit to immune activation (including CD8^+^ T-cell-related biomarkers), validating this strategy [120]. Optimal outcomes require careful dosing and sequencing, often starting with anti-angiogenic therapy to prime the TME for immunotherapy [121]. Future efforts are focused on multitarget agents, biomarker-guided patient selection, and integrating stromal or metabolic modulators to overcome resistance [122].

### Stromal subtype-specific biomarkers

Emerging clinical datasets increasingly support stromal subtype-specific biomarkers as determinants of therapeutic benefit, particularly for immune checkpoint blockade. In metastatic urothelial carcinoma treated with atezolizumab, lack of response was associated with a fibroblast TGF-β signaling signature and an immune-excluded phenotype in which CD8^+^ T cells are retained in the peritumoral stroma rather than infiltrating tumor nests, highlighting a clinically observable stromal program linked to ICI resistance [123]. In HCC, spatial multiomics defined a tumor immune barrier niche composed of CAFs and SPP1^+^ macrophages at the tumor boundary that correlates with ICI efficacy, and independent analyses further implicated POSTN^+^ CAFs as spatial immune response barriers that hinder T-cell infiltration and reduce immunotherapy benefit through an IL-6/STAT3-coupled CAF–macrophage circuit [30]. In colorectal cancer, high-definition spatial mapping showed that the organization of the tumor–stroma boundary stratifies ICI outcomes, where CXCL14^+^ CAF-rich structural barriers are enriched in nonresponders, whereas ICI-responsive boundaries are characterized by higher abundance and interactions of LAMP3^+^ DCs and CXCL13^+^ T cells providing a clinically relevant framework to operationalize stromal niches as response biomarkers [29]. Beyond molecular profiling, low-cost pathology metrics such as tumor–stroma ratio have also been validated as prognostic stromal readouts in colorectal cancer and can serve as a pragmatic companion layer for stratification [124]. Complementing these biomarker advances, several stromal-circuit interventions are being actively tested: for example, the CXCR4 antagonist motixafortide (BL-8040) combined with pembrolizumab and chemotherapy in metastatic PDAC showed immunobiological remodeling (increased intratumoral CD8^+^ effector T cells and reduced MDSCs) with signals of clinical activity, supporting chemokine-axis targeting as a translatable strategy [125]. FAP-directed approaches are also moving forward, including the FAP-targeted immunocytokine simlukafusp alfa (FAP-IL2v) evaluated in basket-trial settings and combination regimens, and FAP-targeted radioligand therapy such as [^177^Lu]Lu-FAP-2286 in advanced solid tumors, both reflecting a shift from CAF ablation to targeted delivery and immune reprogramming [126,127].

Importantly, the field has also generated instructive negative results that clarify resistance mechanisms to stroma-targeted therapies. In HA-high metastatic PDAC, the phase III HALO-301 study showed that adding pegvorhyaluronidase alfa (PEGPH20) to nab-paclitaxel/gemcitabine did not improve overall survival, underscoring that single-marker enrichment (HA-high) may be insufficient when stromal programs are dynamic and redundant [128]. Likewise, Hedgehog-pathway inhibition (e.g., vismodegib) failed to improve outcomes in metastatic PDAC, and earlier clinical experience suggested that indiscriminate stromal depletion can yield counterintuitive biology, consistent with stromal plasticity and compensatory rewiring [129]. More broadly, attempts to cotarget stromal cytokine axes such as TGF-β in late-stage trials have not consistently outperformed existing standards (e.g., bintrafusp alfa versus pembrolizumab in PD-L1-high NSCLC), reinforcing that context-dependent pathway dominance and escape via parallel immunosuppressive circuits are common barriers [130].

### Challenges in clinical translation

The clinical translation of stromal-targeted therapies is hindered by pronounced stromal heterogeneity and a shortage of validated predictive biomarkers. Recent spatial single-cell atlases show that CAF phenotypes and their neighborhood context vary widely across and within tumors and track with outcome, underscoring the need for context-specific strategies [17]. CAF programs also differ by disease: in PDAC, integrative single-cell and spatial maps detail myofibroblastic, inflammatory, and additional fibroblast clusters that dominate desmoplastic niches, whereas triple-negative breast cancers often harbor inflammatory CAF states that couple angiogenesis with chemoresistance in BRCA1-mutant settings [131]. Beyond this static diversity, CAFs exhibit remarkable plasticity under metabolic and paracrine pressures, with nutrient stress or tumor-derived cues maintaining or reprogramming CAF states and reshaping the microenvironment, mechanisms that can blunt single-agent stromal blockade and favor adaptive resistance [132]. Clinically, these dynamics motivate combination or sequential approaches; for example, concurrent blockade of PD-L1 and TGF-β with SHR-1701, alone or with anti-angiogenic therapy, has shown on-treatment biomarker modulation and early activity in refractory solid tumors, supporting multiaxis targeting of stromal–immune circuits [133]. A central barrier remains the absence of robust, prospectively validated selection tools: although CAF phenotypes and gene programs correlate with prognosis, and vascular “normalization” can be monitored with functional imaging, these readouts have not matured into treatment-guiding biomarkers [17]. Emerging noninvasive stromal imaging is promising, as FAP-targeted positron emission tomography/computed tomography (PET/CT) visualizes fibroblast activation in vivo and is beginning to influence staging and restaging decisions, but prospective trials are still small and heterogeneous [134]. Candidate biophysical markers such as collagen architecture or tissue stiffness are quantifiable by second-harmonic generation microscopy and elastography, yet require standardization and validation for routine use [135]. To overcome these hurdles, platforms that couple organotypic “tumor-microenvironment-on-a-chip” models with multiomics readouts, together with artificial intelligence (AI)-driven biomarker discovery from images and molecular data, are being developed to predict stromal therapy benefit and optimize combinations [136]. Trial designs are adapting as well; basket and platform trials aligned to stromal features, plus window-of-opportunity studies with pharmacodynamic endpoints (for example, first-in-human anti-TGF-β programs with paired biopsies), can accelerate biomarker qualification and de-risk clinical translation of stromal-targeted regimens [137].

## TME Stromal Barrier

### Composition and formation of stromal barriers

The stromal barrier is a major obstacle to therapy, integrating mechanical and biochemical impediments that limit transvascular transport and intratumoral diffusion of drugs; recent analyses emphasize dense ECM, elevated interstitial fluid pressure (IFP), and abnormal vasculature as principal determinants of poor delivery [138]. This barrier emerges from continuous, reciprocal signaling between stromal cells and cancer cells that coevolves with disease to create a protective niche favoring progression and treatment resistance [139].

CAFs are central architects of the barrier by overproducing and remodeling ECM macromolecules, particularly fibrillar collagens (types I/III/IV) and hyaluronan, thereby generating a cross-linked, high-density matrix that sterically and hydraulically resists drug penetration [140]. These matrix changes increase stiffness and IFP, further hindering convection and diffusion of therapeutics into tumor parenchyma [141]. Spatially, collagen and hyaluronan accumulate in heterogeneous patterns, with dense peritumoral rims and perivascular niches that correlate with immune exclusion and impaired delivery [140]. CAF activation and ECM deposition are driven by canonical profibrotic pathways, including TGF-β/SMAD, PDGF/PDGFR, and Hedgehog signaling, which are up-regulated across desmoplastic tumors such as PDAC [142].

Tumor vessels exhibit structural and functional abnormalities: endothelial junctional complexes are perturbed (e.g., VE-cadherin and claudin-5 dysregulation), pericyte coverage is reduced or irregular, and basement membranes become discontinuous or abnormally thick, together producing heterogeneous permeability and inefficient, chaotic transport [143]. These aberrations are reinforced by the surrounding matrix and by stromal cross-talk, which destabilize adherens/tight junctions and sustain leakiness and flow heterogeneity [35].

Chronic hypoxia arising from poor perfusion activates HIF-1α programs in fibroblasts and endothelium that increase collagen cross-linking (e.g., LOX/PLOD enzymes), angiogenic signaling, and immunomodulatory ECM remodeling; inflammatory cytokines further sustain CAF activation, creating a self-reinforcing cycle of barrier formation [94].

Multiomics and biophysical studies now link matrix composition/architecture and vascular integrity to drug penetration, motivating combined strategies that normalize ECM mechanics and vessel function (e.g., stiffness/IFP modulation and stromal-targeted delivery) to improve intratumoral exposure [144].

### Quantitative evaluation of barrier penetration

Quantitative assessment of stromal-barrier penetration requires pairing advanced imaging with mechanistic modeling to measure, map, and predict drug transport through dense ECM and abnormal vasculature [145]. Multiphoton intravital microscopy (IVM) resolves drug and nanoparticle kinetics, vascular permeability shifts, and cell–cell interactions at single-cell resolution in living tumors, directly revealing spatial heterogeneity of the “enhanced permeability and retention” effect and routes of intravasation/extravasation [146]. Recent IVM syntheses highlight real-time readouts of stromal–immune dynamics relevant to penetration, including endothelial leak, perivascular flow, and immune cell trafficking that condition barrier function [147].

Dynamic contrast-enhanced magnetic resonance imaging provides noninvasive voxelwise estimates of perfusion and permeability. Pharmacokinetic parameters such as the transfer constant *K*^trans^ and extravascular–extracellular volume fraction *V*_e_ quantify size-selective leakage and regional vascular function and have emerging prognostic/response value in oncology [148].

Super-resolution ultrasound surpasses the diffraction limit to depict microvascular networks and flow dynamics inside stromal-dense regions, offering bedside, repeatable microcirculatory readouts of barrier function [149]. Complementary elastography (ultrasound or magnetic resonance) quantifies tumor and peritumoral stiffness, while stiffness gradients correlate with biological behavior and can stratify treatment response [150]. At the nanoscale, super-resolution microscopy (STORM) and expansion-microscopy variants visualize basement-membrane and interstitial ECM architecture (collagen/laminin) that governs pore size and transport resistance [151].

Imaging-informed transport models integrate vascular density, perfusion, interstitial pressure/flow, and drug properties to simulate spatial–temporal distribution and optimize scheduling, for example, sequencing stroma-modulating agents with chemotherapy to exploit transient normalization [152]. Patient-specific, 3D spatiotemporal models built from clinical imaging have been used to simulate radiopharmaceutical delivery and receptor trafficking within tumors, illustrating how image-derived parameters constrain predictions [153]. Broader image-based predictive frameworks and digital-twin/QSP approaches are emerging to personalize PK/PD, link intratumoral concentrations to efficacy, and prospectively test penetration-enhancing strategies in silico [154].

### Emerging penetration strategies

**Fig. 5.**
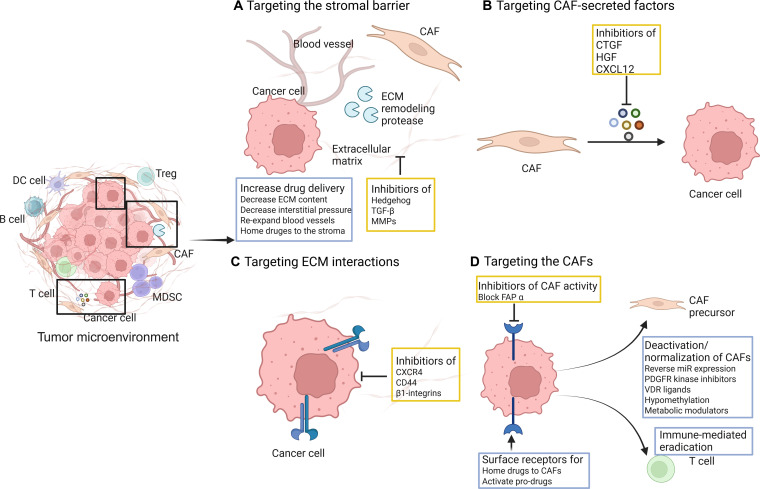
Strategies for targeting CAF-induced protumorigenic effects. (A) Targeting the stromal barrier modulates ECM remodeling, enhances drug delivery, normalizes vessels, etc., via inhibitors (Hedgehog, TGF-β, and MMPs). (B) Targeting CAF-secreted factors (CTGF, HGF, and CXCL12 inhibitors) disrupts protumor paracrine signaling. (C) Targeting ECM interactions blocks cancer cell receptors (CXCR4, CD44, and β1-integrins). (D) Targeting CAFs involves inhibiting CAF activity (FAPα blockade), drug delivery to CAFs, and CAF deactivation/immune eradication.

Multiomics profiling (single-cell, proteomic, and spatial) has clarified how cytokine-programmed CAF ecosystems propel metastasis and, crucially, where and how stromal barriers should be breached to enable therapeutic delivery. Figure 5 illustrates therapeutic strategies to target stromal components in the TME, encompassing approaches to modify the stromal barrier (e.g., inhibiting ECM remodeling proteases or normalizing aberrant blood vessels); block paracrine signals from CAFs such as connective tissue growth factor (CTGF), HGF, and CXCL12; disrupt cancer cell–ECM interactions mediated by molecules like CXCR4, CD44, or β1-integrins; and directly target CAFs, whether by inhibiting their protumor activity (e.g., via FAPα blockade), delivering therapeutics to CAFs through surface receptors, or reconditioning CAF states (including deactivation, normalization, or immune-mediated eradication). Single-cell studies in melanoma identify metastasis-CAF programs that couple SAA3/IL-1β signaling to neutrophil recruitment, directly linking stromal cytokines to premetastatic niche assembly and suggesting IL-1/IL1RAP blockade as a way to soften stromal defenses [105]. In PDAC, endothelial-like CD144^+^ (VE-cadherin) CAFs activate β-catenin/STAT3, drive vasculogenic mimicry, and promote dissemination, nominating endoCAF-directed strategies to interrupt pseudo-vascular conduits that impede uniform drug distribution [155]. In OSCC, myCAFs colocalize with tumor cells at the invasive front and up-regulate cancer-cell stemness programs such as CD44, correlating with nodal spread [156].

Mechanistically, cytokine networks organize these barriers and metastatic routes: TGF-β drives EMT transcriptional circuits (SNAIL/TWIST) and matrix remodeling; IL-6–JAK/STAT3 programs sustain stemness and treatment tolerance; the CXCL12/CXCR4 axis steers tumor-cell homing to stromal sanctuaries; and IL-1β/SAA3 cross-talk enlists myeloid cells to prime premetastatic niches [157]. Clinically oriented stratifiers are emerging: FAP^+^ CAFs drive progression and radioresistance in ESCC, and spatially derived 23-gene invasive-front signatures in OSCC predict lymph-node metastasis and survival, actionable readouts to match penetration tactics to stromal topology [158].

These insights directly inform “penetration” strategies that pair barrier mapping with targeted delivery or barrier-loosening. FAP-targeted nanoparticles and radiopharmaceuticals deliver stromal-lytic payloads to FAP-rich regions. Collagenase-decorated nanocarriers or biomaterials temporarily reduce matrix stiffness and improve intratumoral dispersion. CXCR4 antagonists disrupt CXCL12-guided tumor homing to protected niches. CAF-IL1RAP neutralization dampens neutrophil-priming cytokine loops that stiffen the premetastatic niche [159].

## Current Challenges and Future Perspectives

### Technical challenges

Integrating transcriptomic, epigenomic, proteomic, and spatial modalities is intrinsically difficult because each technology has distinct dynamic ranges, dropout/error structures, and assay-specific biases, which amplify when data are combined; recent reviews and methods papers document that platform heterogeneity and batch effects can obscure stromal signals and hinder subtype discovery if not rigorously addressed [160]. Even within scRNA-seq, droplet (e.g., 10×) and plate-based (e.g., Smart-seq) chemistries differ in sensitivity and noise, and cross-vendor droplet systems vary in capture efficacy and gene-detection capacity, complicating downstream alignment to proteomic or spatial readouts [161].

State-of-the-art batch-correction methods must remove technical variation yet preserve subtle biological gradients that define stromal phenotypes. Mutual nearest-neighbor families (fastMNN/Seurat v5) remain widely used but can distort structure or become order-dependent as batches scale, motivating optimal-transport and semisupervised variants and principled tests of “alignability” before integration [162]. Large benchmarks further show that choices in correction can reshape phenotypes and that evaluation should quantify both batch removal and biology retention across tasks (label transfer, unseen-population detection) [163]. Deep generative models (e.g., scVI/scANVI-style architectures) and new multiomics aligners (e.g., scMODAL) improve vertical and diagonal integration, but require careful tuning and substantial computation, especially for atlas-scale cohorts [164].

A major bottleneck is the absence of standardized, consensus pipelines for stromal cell identification that reconcile discrete “marker lists” with continuous phenotypic spectra. Semisupervised tools for cell-type harmonization across studies (e.g., CellHint) and population-level learners (e.g., scPoli) help stabilize labels and preserve biological variance during mapping, yet community standards for subtype criteria remain uneven across tissues and diseases [165]. Reference-based integration is emerging as a practical path: Seurat v5 “bridge integration” uses multiome data to align RNA/ATAC and other modalities, while scArches enables federated updating of atlases without raw-data sharing [166]. Spatial data add further complexity (registration, multislice alignment, and cross-platform normalization); recent surveys and algorithms emphasize rigorous validation across sections and instruments [167].

Scalability and accessibility are nontrivial. Atlas-level resources (e.g., HCA and HuBMAP) are pushing standards for data models and sharing, while software ecosystems adopt common containers and file formats, such as SpatialData (scverse) and OME-NGFF (OME-Zarr), to make multimodal spatial images and tables interoperable [168,169]. Visualization and exploration at scale remain challenging; new web frameworks (e.g., Vitessce) aim to render multi-million-cell, multimodal atlases interactively for quality control and hypothesis generation [170].

Looking forward, community benchmarks and curated tumor atlases should serve as reference scaffolds to validate methods and stabilize labels; exemplars include multimodal/diagonal integration benchmarks and learning-based pipelines that map dynamic contrast-enhanced/spatial time series directly to biologically meaningful parameters. Machine learning solutions, multimodal autoencoders for latent cross-modal representations and graph neural networks that encode tissue topology, are particularly promising for stromal biology, provided their outputs are coupled to transparent uncertainty and robust cross-study validation [171].

### Biological challenges

Stromal cells, especially CAFs, are highly plastic, toggling between inflammatory iCAF and myofibroblastic myCAF programs under opposing IL-1–JAK/STAT and TGF-β–SMAD cues, with mechanical inputs further biasing state transitions. Such adaptability enables escape from single-axis therapies and fosters treatment resistance [172]. Intercellular cross-talk is multilayered: direct contacts and paracrine ligand–receptor signaling are complemented by CAF-derived extracellular vesicles that reprogram tumor and immune cells, and by tunneling nanotubes that shuttle organelles and metabolites to support invasion and drug tolerance [173]. The functional output of a given pathway (for example, TGF-β between CAFs and cancer cells) depends critically on spatial context, timing, and cellular composition, motivating computational frameworks that integrate single-cell and spatial measurements to infer context-aware communication [174].

Substantial spatial heterogeneity compounds these challenges. i/myCAF mixes and specialized CAF niches differ between invasive fronts, tumor cores, and perivascular hubs that nurture stemness and dissemination; recent spatial atlases in solid tumors (including PDAC and NSCLC) map FAP^+^ CAF clusters and POSTN^+^ CAF neighborhoods linked to immunosuppressive myeloid aggregates [53]. Yet, current single-cell and spatial transcriptomic platforms variably trade sensitivity, resolution, and throughput, and cross-platform integration remains nontrivial, risking loss of subtle stromal gradients without rigorous benchmarking and normalization [175].

Temporal dynamics introduce an additional layer of complexity. Longitudinal single-cell and spatial studies under immunotherapy reveal evolving stromal–immune circuits that correlate with resistance states, yet serial patient sampling is logistically difficult and only partially recapitulated by preclinical models [176]. Progress will hinge on improved lineage tracing to causally track stromal fate transitions (e.g., CRISPR/barcode systems and prime-editing recorders), computational lineage inference from single-cell data, and physiologically faithful stromal–organoid assembloids that enable controlled perturbation [177]. Together, combining high-resolution spatial mapping, causal lineage tools, and mechanistic models of ligand–receptor exchange is essential to decode stromal signaling and plasticity, and to design therapies that anticipate, rather than react to, adaptive resistance [178].

### Clinical challenges

Translating stromal biology into clinically useful biomarkers remains difficult because stromal programs are heterogeneous across tumors and evolve with therapy. Unlike relatively stable tumor mutations, stromal phenotypes are plastic and microenvironment-conditioned, myofibroblastic programs dominate desmoplastic PDAC whereas inflammatory CAF states are frequent in basal/TNBC and squamous malignancies, and rCAF subsets can restrain disease, mandating disease-specific validation [131]. Therapy further reshapes stroma: on-treatment single-cell/spatial profiling reveals trajectory switches under immunoradiotherapy, underscoring the need for serial assessment rather than one-time pretreatment stratification [54].

Technical constraints also limit deployment. Routine IHC/RNA assays struggle with overlapping markers, low stromal abundance, and sampling bias from spatial heterogeneity, such as spatial studies that demonstrate block-like regional divergence within single tumors and discordant biomarker status across biopsies [179]. While spatial transcriptomics and multiplexed tissue imaging (e.g., imaging mass cytometry) offer richer readouts, platform variability, FFPE compatibility, and the absence of standardized pipelines continue to impede clinical adoption [180].

Promising measurement strategies are emerging. FAP-PET/CT (e.g., NCT04023240, NCT05262855, and NCT05641896) visualizes fibroblast activation noninvasively and is moving toward procedural standards and early prospective data, enabling whole-body stromal burden mapping for staging and restaging [181,182]. Circulating ECM turnover fragments (e.g., PRO-C3/C3M and related neo-epitopes) quantify desmoplasia and associate with prognosis across cancers, offering scalable, repeatable liquid-biopsy surrogates of stromal activity [183]. CAF-derived extracellular vesicles are increasingly detectable in plasma and carry subtype-linked cargos (miRNAs/proteins) with diagnostic and prognostic utility, though clinical specificity remains to be proven [184]. In parallel, computational pathology can infer stromal states from standard H&E, with deep-learning models imputing transcriptomic programs and predicting outcomes, providing a practical route to deploy stromal signatures at scale [185].

Progress will require large, well-annotated biobanks with serial sampling to capture temporal plasticity; harmonized analysis standards for spatial and multiplex assays; and prospective trials that embed stromal biomarkers into eligibility, stratification, and on-treatment decision rules [186]. Reference-guided integration of multiomics (single-cell plus spatial) can stabilize subtype labels across cohorts, while liquid- and image-based readouts (FAP-PET/CT and ECM fragments) provide repeatable, system-level monitoring, together enabling stromal, subtype-aware patient selection beyond purely tumor-centric paradigms.

### Future directions

Future work should integrate single-cell, spatial multiomics with advanced lineage tracing to resolve stromal origins, plasticity, and fate decisions in situ. Recent advances in CRISPR barcoding, including dual-nuclease Cas9/Cas12a recorders with improved information density, enable linkage of lineage history to cell state at single-cell resolution, while emerging spatiotemporal lineage-tracing frameworks map how clones expand across tissue architecture during tumor growth and metastasis. These tools, coupled with multiomic lineage inference, provide a route to reconstruct developmental trajectories of stromal subpopulations and to pinpoint niche-encoded cues that license protumorigenic functions [187].

Therapeutically, the field should pivot from indiscriminate stromal depletion to precision modulation of pathogenic subtypes while sparing homeostatic stroma. Next-generation effectors include dual-specificity/bispecific ADCs (enhanced avidity and internalization), FAP-directed conjugates/small-molecule drug conjugates for CAF-rich niches, and logic-gated nanotherapies that execute AND-gate delivery or editing only within tumor-conditioned microenvironments. In parallel, epigenetic modulators that reprogram CAF states, such as class I HDAC inhibition curbing myofibroblastic activation in PDAC, or epigenetic payloads delivered by ADCs offer selective rewiring of stromal programs [188,189].

AI-driven stromal subtype prediction models represent a timely future direction for TME research, as they can translate routine imaging into clinically actionable stromal phenotypes for risk stratification and trial design [190,191]. In computational pathology, deep learning can segment tumor epithelium and stroma and compute the tumor–stroma ratio (TSR) automatically, and AI-quantified TSR has shown independent prognostic value across resectable colorectal cancer and pancreatic cancer cohorts [192,193]. Beyond bulk TSR, models that score stromal reaction phenotypes (e.g., fibrosis, cellularity, and orientation at the tumor-stroma interface) enable large-scale, reproducible assessment and can be linked to prognosis-associated molecular signatures [194]. At a higher level of abstraction, imaging-based deep learning has been used to noninvasively classify immune and stroma microenvironment states from CT scans and predict outcomes and treatment benefit, providing a feasible route for longitudinal monitoring of stromal dynamics during therapy [191]. Weakly supervised frameworks such as HistoTME further suggest that WSIs can predict cell type-specific TME signatures (including fibroblast/CAF programs) and immunotherapy response, which could be adapted to output stromal subtype probabilities in clinical workflows [195]. Future multimodal approaches that combine WSI pathomics, radiomics, and spatial transcriptomic supervision may yield interpretable stromal subtype maps to guide patient selection for stroma-targeting or immunomodulatory strategies and to enrich biomarker-driven trials [196].

Multimodal combination strategies should be timed by computational modeling to exploit transient windows of microenvironment “normalization”. For matrix-dominated barriers, pan-lysyl-oxidase inhibition (e.g., PXS-5505) reduces collagen cross-linking and improves chemotherapy penetration in preclinical PDAC, while anti-angiogenic dosing can open a short-lived vascular-normalization window that improves perfusion and immunotherapy delivery [90]. Moreover, TME-responsive carriers (pH/MMP-activated) can further concentrate payloads spatiotemporally [197].

Clinical translation will benefit from real-time, biomarker-driven trial designs and adaptive regimens that adjust to on-treatment stromal states, supported by emerging digital-twin and pharmacometric platforms that fuse imaging, spatial-omic, and liquid-biopsy data to prospectively simulate scheduling and combinations [198]. Basket/platform trials anchored to stromal features, coupled with iterative stratification using whole-body stromal imaging and circulating ECM/vesicle readouts, can operationalize stromal reprogramming in routine care [137].

## Conclusion

Single-cell and spatial multiomics have transformed stromal biology from a static framework into a dynamic, spatiotemporal system that actively governs tumor progression, immune evasion, drug delivery, and metastatic spread. By resolving conserved stromal archetypes (e.g., myCAFs, iCAFs, endothelial, and pericyte subtypes) and their cytokine circuits (TGF-β, IL-6, and CXCL12/CXCR4), these technologies reveal how ECM architecture, vascular dysfunction, and stromal–immune neighborhoods jointly create therapy-refractory niches. Quantitative imaging and pharmacokinetic modeling now link matrix mechanics and vascular integrity to intratumoral drug exposure, clarifying why monotherapies underperform and why rational combinations, normalizing vessels or matrix while relieving immunosuppression, produce durable benefit only within well-timed therapeutic windows.

Future progress will hinge on integrating lineage-resolved, spatial multiomics with standardizable analytics to decode stromal plasticity in situ and to nominate subtype-specific, low-toxicity interventions. Therapeutically, the field should pivot from broad depletion to precision modulation of pathogenic phenotypes using next-generation modalities, FAP-directed conjugates and radiopharmaceuticals, logic-gated nanotherapies responsive to TME cues, and epigenetic or pathway modulators that reprogram CAF states, embedded within multimodal sequences that first normalize the barrier (e.g., TGF-β or LOX axis modulation and vascular normalization) and then deliver targeted or immune therapies. Clinically, real-time, biomarker-guided and adaptive trial designs, leveraging stromal imaging (e.g., FAP-PET/CT), liquid-biopsy ECM fragments, and computational “digital-twin” scheduling, should enable stromal subtype-aware patient selection and dosing, moving oncology beyond tumor-centric paradigms toward reproducible, spatially precise stromal reprogramming.
